# Oral Hydrogen-Rich Water Alleviates Oxalate-Induced Kidney Injury by Suppressing Oxidative Stress, Inflammation, and Fibrosis

**DOI:** 10.3389/fmed.2021.713536

**Published:** 2021-08-19

**Authors:** Yachen Si, Lulu Liu, Jin Cheng, Tingting Zhao, Qi Zhou, Jianpeng Yu, Wei Chen, Jiarong Ding, Xuejun Sun, Hongtao Lu, Zhiyong Guo

**Affiliations:** ^1^Department of Nephrology, Shanghai Changhai Hospital, Naval Medical University, Shanghai, China; ^2^Internal Medicine III (Nephrology and Endocrinology), Naval Medical Center, Naval Medical University, Shanghai, China; ^3^Department of Naval Medicine, Naval Medical University, Shanghai, China; ^4^Center of Hydrogen Science, Shanghai Jiao Tong University, Shanghai, China

**Keywords:** hydrogen-rich water, oxalate-induced kidney injury, oxidative stress, inflammation, fibrosis

## Abstract

**Objective:** To explore the theraputic effects and potential mechanisms of hydrogen-rich water (HRW) against oxalate-induced kidney injury.

**Methods:** The mouse model of Calcium oxalate (CaOx) crystallization was established by feeding a soluble oxalate diet. Crystal deposition, tubular injury, fibrosis and reactive oxygen species (ROS) production in kidneys were examined by histology. Serum indexes of renal injury, inflammation and oxidative stress were detected by commercial kits. RNA sequencing (RNA-seq) was performed to screen potential pathways and the expressions of key molecules in these pathways were determined by western blotting and immunohistochemistry.

**Results:** Crystal deposition, tubular injury, fibrosis and increased ROS production in kidneys of mice induced by oxalate diet were improved with HRW administration. The indexes of renal injury, inflammation and oxidative stress in serum of mice were upregulated by oxalate diet, which were reduced by HRW. A total of 3,566 differential genes were screened by RNA-seq and these genes were analyzed by pathway enrichment and PI3K/AKT, NF-κB, and TGF-β pathways were selected for further verification. The expressions of molecules related to PI3K-AKT pathway (PI3K, AKT, and p-AKT), NF-κB pathway (NF-κB p65, p- NF-κB p65, NLRP3, and IL-1β) and TGF-β pathway (TGF-β, TGF-βRI, TGF-βRII, p-Smad2, and p-Smad3) in renal tissues were increased by oxalate diet, which were reduced by HRW administration.

**Conclusion:** HRW may alleviate oxalate-induced kidney injury with its anti-oxidative, anti-inflammatory and anti-fibrotic effects via inhibiting PI3K/AKT, NF-κB, and TGF-β pathways.

## Introduction

The prevalence of nephrolithiasis has been increasing worldwide and about one in 17 adults suffer from kidney stones in China (1, 2). Stone formers are at risk of developing chronic kidney disease (CKD) and kidney failure, which further damages the quality of life and aggravates the financial burden (3, 4). Therefore, it is of great significance to find potential therapy for kidney stones.

Calcium oxalate (CaOx), as the most common constituent of kidney stones, can interact with renal tubular epithelial cells and induce oxidative stress, inflammation and fibrosis, further promoting tubular cell injury (5). Oxidative stress is associated with excessive production of reactive oxygen species (ROS) in response to CaOx crystals, which can be ameliorated by antioxidants and free radical scavengers (6). ROS can activate inflammasome and various transcription factors, leading to the production of inflammatory cytokines. Excessive oxidative stress and inflammation not only enhance the deposition and retention of CaOx crystals in tubular cells, but also result in the development of fibrosis (7). Thus, it is urgent to explore an effective treatment with anti-oxidative, anti-inflammatory and anti-fibrotic effects in this field.

Previous basic and clinical research has revealed that molecular hydrogen exerts multiple biological effects involving anti-oxidation, anti-inflammation, anti-fibrosis and anti-apoptosis in many diseases of different systems (8). More recently, our group reported that inhalation of hydrogen gas can alleviate glyoxylate-induced CaOx deposition and metabolic disturbance of blood and urine in mice (9, 10). Drinking hydrogen-rich water (HRW) represents an alternative mode of delivery of molecular hydrogen. The primary advantages of HRW are that it is a portable, easily administered, and safe means of delivering hydrogen. Therefore, the potential effects of HRW on oxalate-induced kidney injury need further research.

Previous studies indicated that feeding a soluble oxalate diet represents a successful mouse model of CaOx crystallization, which has the characteristics of CKD [PMID: (11, 12)]. In this study, oxalate diet induced kidney injury was established to determine the theraputic effects of HRW. RNA sequencing (RNA-seq), as a technique for detecting the expression level of the transcriptome, has the advantages of quantitative accuracy, high levels of reproducibility and a wide detection range (13). In the present study, RNA-seq analysis was performed to detect the expression of mRNA profiles in the renal tissues of mice fed normal diet and oxalate diet. Subsequently, the potential pathways associated with oxalate diet induced kidney injury were analyzed and screened. The aim of the study was to provide novel insights into the potential mechanisms of HRW against oxalate-induced kidney injury.

## Materials and Methods

### Animal Experiments

Male ICR mice aged 6–7 weeks and weighed 25–30 g were purchased from Shanghai Jiesijie Lab Animal Co., Ltd. (Shanghai, China) and allowed to acclimatize to the lab environment for 1 week. The animal protocols were approved by Committee on Ethic of Medical Research, Naval Medical University (registration number: NMUMREC-2021-006). The mice (*n* = 24) were randomly assigned into three groups: Group 1 (ND group, *n* = 8) were given normal diet and regular water for 14 days; Group 2 (OD group, *n* = 8) were given oxalate diet and regular water for 14 days; Group 3 (OH group, *n* = 8) were given oxalate diet and HRW for 14 days. Normal diet (SLACOM, Shanghai, China) was conventional breeding feed for mice (P1101F). A high oxalate diet (Harlan, Madison, WI, USA) was prepared by adding sodium oxalate (50 μmoles/g) to a virtually calcium-free diet (TD.95027). The HRW (hydrogen concentration ≥ 2.5 ppm) was produced by a hydrogen water apparatus (Nanobubble, Shanghai, China). To reduce the loss of hydrogen, HRW was stored in aluminum bags and replaced every 8 h. Regular water was generated by degassing HRW by gentle stirring for 24 h.

At the end of the experiment, the mice were all anesthetized. Blood was collected and serum was obtained after centrifugation at 3,000 rpm for 10 min and stored at −80°C. After *in situ* cardio-perfusion, the right kidneys were immediately removed and stored at −80°C. Next, the left kidneys were removed and fixed in 10% buffered formalin.

### Determination of Biochemical Indexes

Mice serum creatinine (SCr), blood urea nitrogen (BUN), Malondialdehyde (MDA), Superoxide dismutase (SOD), Glutathione peroxidase (GSH-Px) and Catalase (CAT) were measured by commercial kits (Jiancheng Bioengineering, Nanjing, Jiangsu, China). The levels of Kidney injury molecule-1 (KIM-1), Neutrophil gelatinase-associated lipocalin (NGAL), Tumor necrosis factor-α (TNF-α) and Interleukin-1β (IL-1β) in serum of mice were detected by corresponding kits (Jingmei Biotechnology, Yancheng, Jiangsu, China).

### Renal Histology

The kidney samples that fixed in 10% buffered formalin were embedded in paraffin and sectioned at a thickness of 4 μm and submitted to hematoxylin and eosin (HE) staining. In the HE-stained sections, tubular damage score was assigned semiquantitatively on a scale of 0–5: 0 = normal histology; 1 = degeneration only without necrosis; and 2~5 = < 25, < 50, < 75, and > 75%, respectively, of epithelial cells showing necrosis, degeneration, regeneration, tubular dilatation, protein casts, and interstitial lymphocytic infiltration. The renal crystals were observed using a polarizing microscope (Nikon, Chiyoda, Tokyo, Japan). For pizzolato staining, silver nitrate-hydrogen peroxide solution (equal quantities of 5% silver nitrate and 30% Hydrogen peroxide) were added to the sections. After exposure under the ultraviolet lamp for 30 min, the sections were rinsed thoroughly with distilled water. Then, the sections were counterstained in nuclear fast red solution for 5 min and dehydrated conventionally. The areas of interstitial fibrosis were detected using Masson trichrome staining, which was stained dark blue.

The kidney samples that stored at −80°C were prepared into frozen slices to detect renal reactive oxygen species (ROS) production. The sections were washed twice with phosphate buffer saline (PBS) and incubated with 20 μM dihydroethidium (DHE; Beyotime Biotechnology, Haimen, Jiangsu, China) diluted in 2 mL serum-free Dulbecco's modified Eagle's medium (DMEM) without light at 37°C. After 30 min incubation, the sections were washed three times with PBS, and fluorescence microscopy was used to observe the red fluorescent images.

### Immunohistochemistry Analysis

The sections were incubated with citrate antigen retrieval solution for 20 min at 95°C. Thereafter, the sections were incubated with primary antibodies against osteopontin (OPN; 1:100, Immunoway, Newark, DE, USA), cluster of differentiation 44 (CD44; 1:100, Immunoway, Newark, DE, USA), α-smooth muscle actin (α-SMA; 1:100, Cloud-Clone, Wuhan, Hubei, China), fibronectin (FN; 1:50, Cloud-Clone, Wuhan, Hubei, China), collagen IV (COL IV; 1:100, Cloud-Clone, Wuhan, Hubei, China), Phosphatidylinositol 3-kinase (PI3K; 1:100, Cell Signaling Technology, Danvers, MA, USA), p-serine/threonine kinase (p-AKT; 1:100, Cell Signaling Technology, Danvers, MA, USA), nuclear factor-κB (NF-κB) p65 (1:500, Cell Signaling Technology, Danvers, MA, USA), p-NF-κB p65 (1:100, Santa Cruz Biotechnology, Dallas, TX, USA), NACHT, LRR and PYD domain-containing protein 3 (NLRP3; 1:200, Cell Signaling Technology, Danvers, MA, USA), IL-1β (1:100, Cell Signaling Technology, Danvers, MA, USA), transforming growth factor-β (TGF-β; 1:50, Cloud-Clone, Wuhan, Hubei, China), p-Recombinant Mothers Against Decapentaplegic Homolog 2 (p-Smad2; 1:100, Cell Signaling Technology, Danvers, MA, USA) and p-Smad3 (1:100, Cell Signaling Technology, Danvers, MA, USA) overnight at 4°C and then with HRP-tagged goat anti-rabbit antibody (1:200, Proteintech, Rosemont, IL, USA) at 37°C for 50 min. Finally, the sections were stained with diaminobenzidine and counter-stained with hematoxylin.

### RNA-seq Analysis

Total RNA was extracted from the renal tissues of mice in the ND group and the OD group using TRIzol reagent (Invitrogen, Carlsbad, CA, USA). Then, RNA integrity, purity and concentration were analyzed with Qubit RNA assay kit (Thermo Fisher Scientific, Waltham, MA, USA). After that, the mRNA was concentrated using magnetic beads with Oligo (dT) and randomly broken into short fragments. Double-stranded cDNA was synthesized and purified from template mRNA with AMPure XP beads. The cDNA end sequences were completed *via* End Repair Mix, and poly A tails and sequencing connectors were added. The connecting products were purified and fragment size was separated, and cDNA library amplification was performed using PCR technology. Finally, sequencing was performed with a HiSeq Illumina System (Illumina, San Diego, CA, USA).

Visual evaluation of raw data quality was performed with fastQC (https://www.bioinformatics.babraham.ac.uk/projects/fastqc/). Then, Trimmomatic (http://www.usadellab.org/cms/?page=trimmomatic) was used to process and filter the raw data. The TPM (Transcripts Per Million) method was used to determine the gene expression level. The DESeq algorithm was applied to filter the differentially expressed genes (DEGs) under the criteria of *q*-value (corrected *p*-value) < 0.05, |log_2_FoldChange| > 1 and mean TPM of at least one group > 5.

### Bioinformatics Analysis

Kyoto Encyclopedia of Genes and Genomes (KEGG) pathway analysis was used to determine the significant pathways of the differential genes according to clusterProfiler (http://www.bioconductor.org/packages/release/bioc/html/clusterProfiler.html). The STRING (https://www.string-db.org/) database was utilized to construct the protein-protein interaction (PPI) network. Cytoscape (https://cytoscape.org/) was used for visual exploration of interactive networks. The Cytoscape plug-in cytoHubba (http://apps.cytoscape.org/apps/cytohubba) was used to identify the hub genes.

### Western Blotting Analysis

Proteins from the renal tissues were extracted in lysis buffer (KeyGEN, Nanjing, Jiangsu, China) supplemented with phenylmethylsulfonyl fluoride (PMSF), phosphatase inhibitors, and protease inhibitors. Equal amounts of the protein samples were loaded on 10% sodium dodecyl sulfate-polyacrylamide gel electrophoresis (SDS-PAGE) and then transferred onto a nitrocellulose filter membrane (GE Healthcare Life Sciences, Little Chalfont, UK). After blocking in 5% bovine serum for 2 h, the membranes were incubated at 4°C overnight with primary antibodies against nicotinamide adenine dinucleotide phosphate (NADPH) oxidase 2 (NOX2; 1:1,000, Abways, Shanghai, China), NOX4 (1:1,000, Abcam, Cambridge, UK), α-SMA (1:1,000), FN (1:1,000), COL IV (1:1,000), PI3K (1:1,000), AKT (1:1,000, Cell Signaling Technology, Danvers, MA, USA), p-AKT (1:1,000), NF-κB p65 (1:1,000), p- NF-κB p65 (1:1,000), NLRP3 (1:1,000), IL-1β (1:1,000), TGF-β (1:1,000), TGF-β receptor I (TGF-βRI; 1:1,000, Abways, Shanghai, China), TGF-βRII (1:1,000, Abways, Shanghai, China), p-Smad2 (1:1,000), p-Smad3 (1:1,000), GAPDH (1:1,000, Cell Signaling Technology, Danvers, MA, USA) and β-actin (1:1,000, Cell Signaling Technology, Danvers, MA, USA). Then the membranes were washed three times with Tween 20/Tris-buffered saline (TBST) and incubated with the secondary antibody (LI-COR Biosciences, Lincoln, NE, USA) for 2 h at room temperature. After washing three times with TBST, the fluorescence signal of the secondary antibody could be detected by the Odyssey Fluorescence Imaging System (LI-COR Biosciences, Lincoln, NE, USA).

### Statistical Analysis

Experimental data were expressed as mean ± standard error of the mean (SEM) and were analyzed with SPSS 22.0 statistical software (SPSS Inc., Chicago, IL, USA) using a one-way ANOVA followed by Tukey's post-test. A *p*-value of <0.05 was considered as indicating a statistically significant difference.

## Results

### HRW Administration Ameliorates Oxalate-Induced Crystal Deposition and Renal Injury in Mice

**Figure 1:**
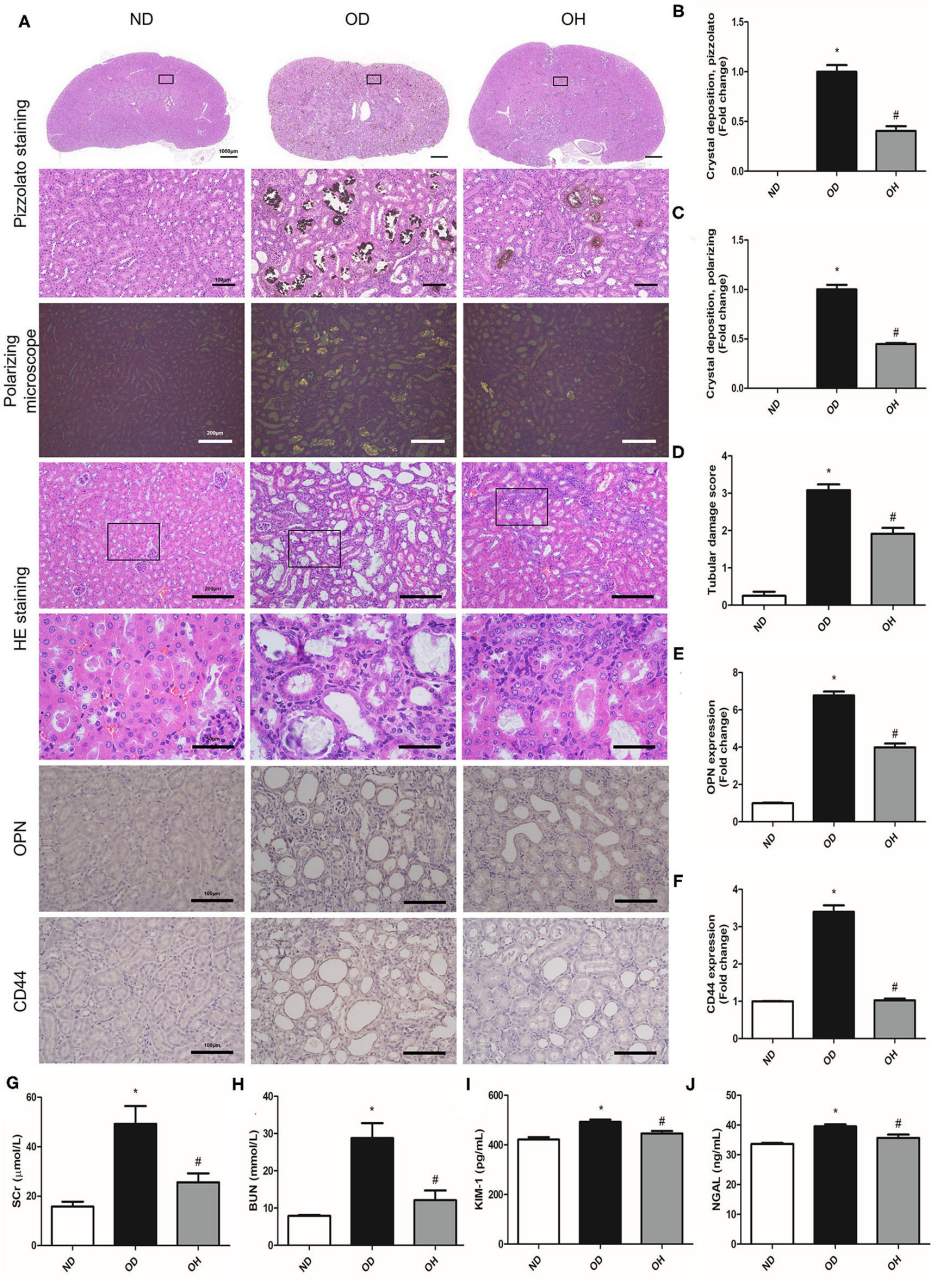
HRW administration ameliorates oxalate-induced crystal deposition and renal injury in mice. **(A)** The CaOx crystal deposition in kidneys of mice was detected by pizzolato staining (Scale bar = 1,000 and 100 μm) and polarization microscopy (Scale bar = 200 μm); The renal histological changes in mice were observed by HE staining (Scale bar = 200 and 50 μm); The protein expressions of OPN and CD44 in kidneys of mice were detected by immunohistochemistry (Scale bar = 100 μm). **(B–F)** Corresponding semiquantitative analysis of pizzolato staining, polarization microscopy, HE staining and OPN and CD44 immunohistochemistry staining. **(G–J)** The levels of SCr, BUN, KIM-1, and NGAL in serum of mice were determined by corresponding kits. The results were expressed as mean ± SEM. Statistical comparisons were performed using a NewmanKeuls test (^*^p < 0.05 vs. ND group, ^#^*p* < 0.05 vs. OD group).

By performing pizzolato staining and polarization microscopy of kidney sections, there found extensive CaOx crystal deposition in kidneys of mice fed a diet high in soluble oxalate for 14 days. It was demonstrated that there were much fewer renal CaOx crystals with HRW administration (Figures 1A–C). HE staining showed that obvious renal changes, such as tubular dilation, degeneration and necrosis of epithelial cells, and inflammatory cells infiltration in renal interstitium, were examined in the OD group, whereby HRW administration was able to attenuate these histological damages (Figures 1A,D). The expressions of OPN and CD44 in kidneys of mice were increased in the OD group as compared to the ND group, which were downregulated with HRW administration (Figures 1A,E,F). Furthermore, compared to the ND group, the levels of SCr, BUN, KIM-1, and NGAL in serum of mice were significantly elevated in the OD group. However, the contents of these biomarkers were significantly reduced with HRW administration (Figures 1G–J).

### HRW Administration Improves Oxalate-Induced Inflammation and Oxidative Stress in Mice

**Figure 2:**
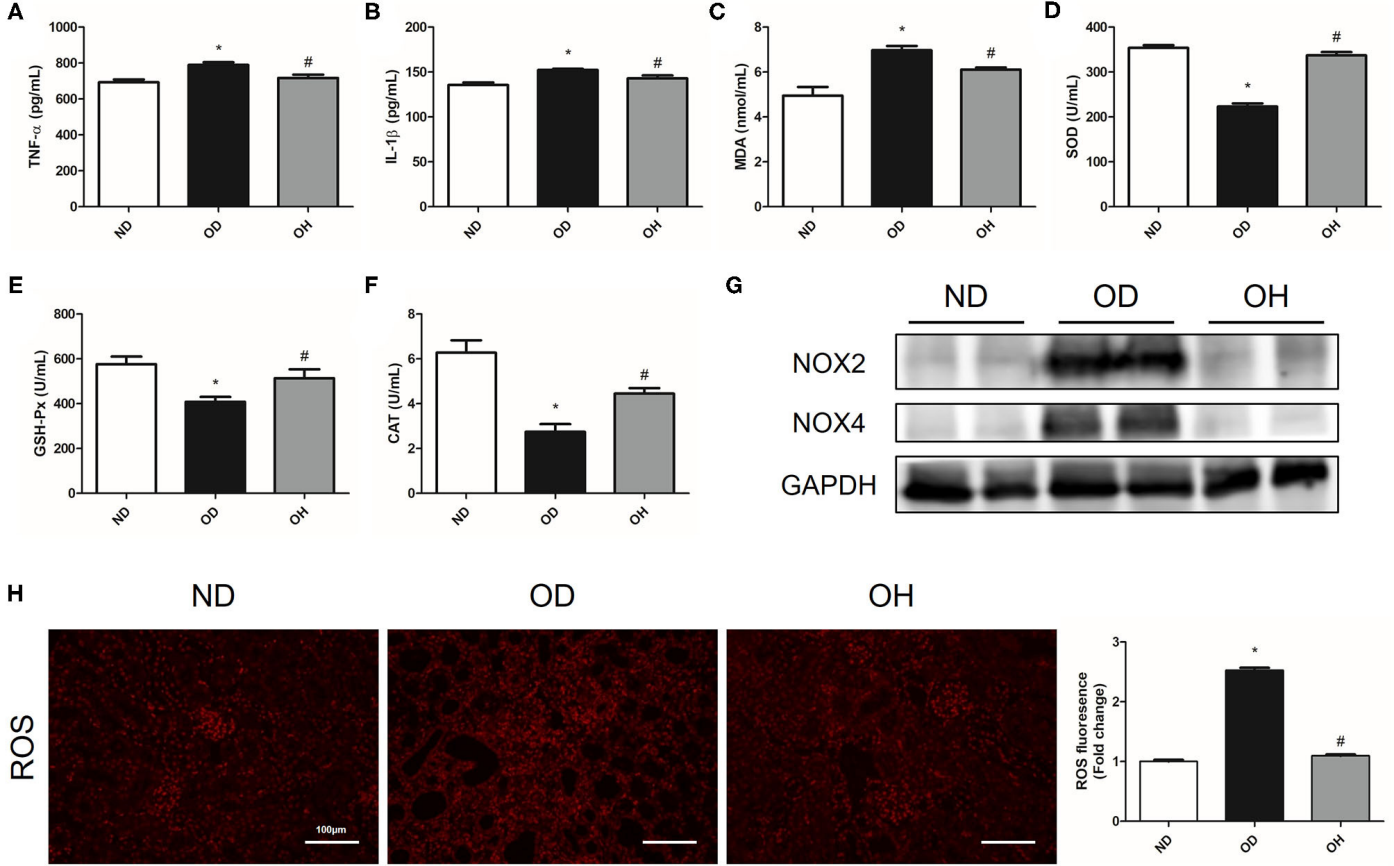
HRW administration improves oxalate-induced inflammation and oxidative stress in mice. **(A–F)** The levels of TNF-α, IL-1β, MDA, SOD, GSH-Px and CAT in serum of mice were determined by corresponding kits. **(G)** The protein expressions of NOX2 and NOX4 in kidneys of mice were detected by western blotting. **(H)** ROS production in the renal tissues of mice was detected by immunofluorescence (Scale bar = 100 μm). The results were expressed as mean ± SEM. Statistical comparisons were performed using a NewmanKeuls test (*p < 0.05 vs. ND group, ^#^*p* < 0.05 vs. OD group).

About the inflammation indexes, the levels of TNF-α and IL-1β in serum of mice were higher in the OD group than the ND group, which were reduced by HRW administration (Figures 2A,B). When it comes to oxidative stress markers, there were significantly increased MDA content as well as decreased SOD, GAH-Px and CAT activity in serum of mice in the OD group compared to those in the ND group. But the levels of these markers were reversed in the OH group (Figures 2C–F). Then, the protein expressions of NOX2 and NOX4 in kidneys of mice were analyzed. As compared to the ND group, the expressions of NOX2 and NOX4 were enhanced in the OD group, which were downregulated by HRW administration (Figure 2G). In addition, increased renal ROS production from high oxalate diet was mitigated by HRW administration (Figure 2H).

### HRW Administration Alleviates Oxalate-Induced Renal Fibrosis in Mice

**Figure 3:**
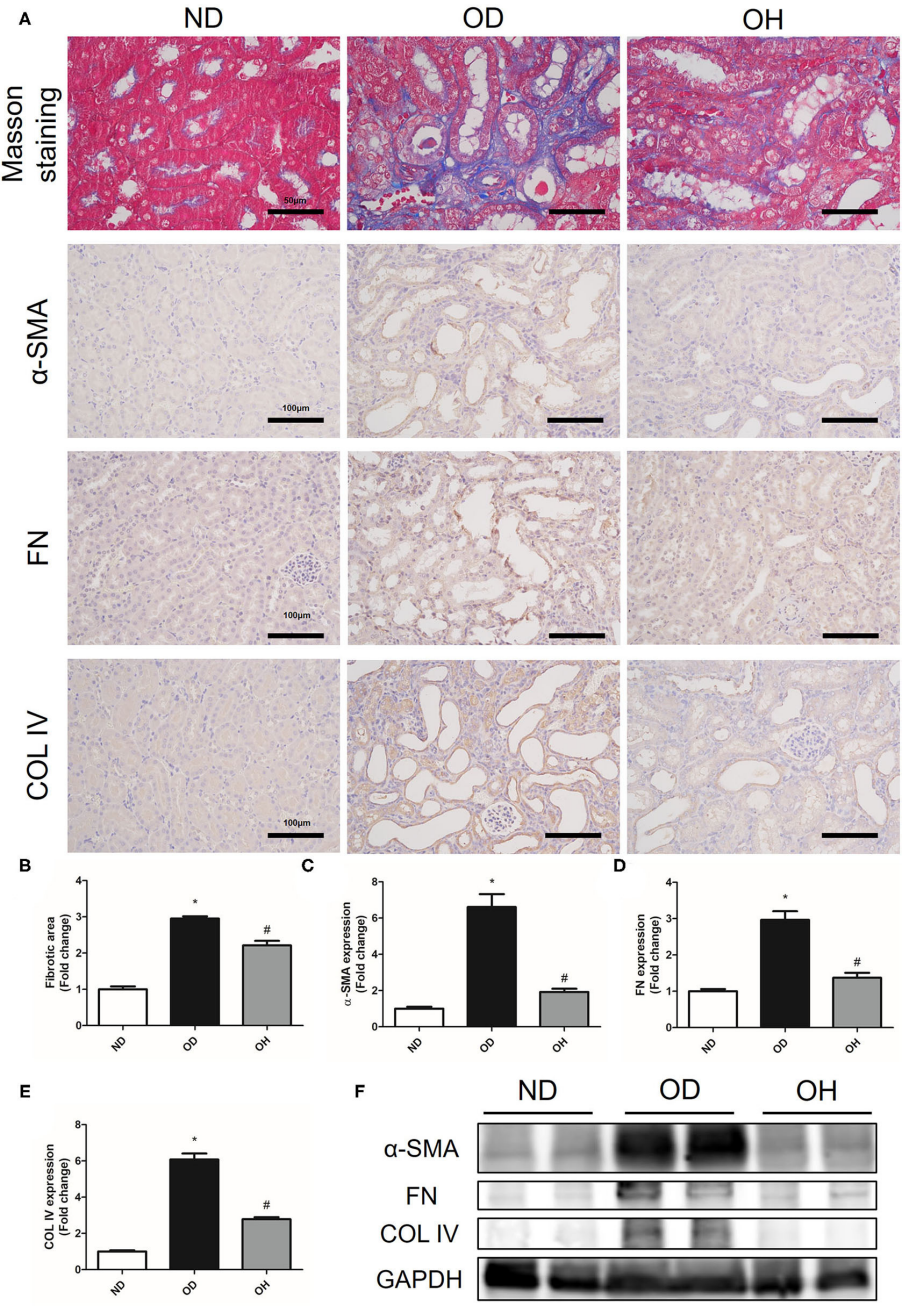
HRW administration alleviates oxalate-induced renal fibrosis in mice. **(A)** The degree of renal collagen was assessed with Masson trichrome staining (Scale bar = 50 μm); The protein expressions of α-SMA, FN, and COL IV in kidneys of mice were detected by immunohistochemistry (Scale bar = 100 μm). **(B–E)** Corresponding semiquantitative analysis of Masson trichrome staining and α-SMA, FN and COL IV immunohistochemistry staining. **(F)** The protein expressions of α-SMA, FN and COL IV in kidneys of mice were detected by western blotting. The results were expressed as mean ± SEM. Statistical comparisons were performed using a NewmanKeuls test (^*^p < 0.05 vs. ND group, ^#^*p* < 0.05 vs. OD group).

The relative content of the degree of renal collagen was assessed with Masson trichrome staining so as to reflect the degree of renal fibrosis. It was demonstrated that renal interstitial fibrosis caused by high oxalate diet was improved by HRW administration (Figures 3A,B). Moreover, immunohistochemistry showed that the expressions of α-SMA, FN, and COL IV in kidneys of mice were increased in the OD group as compared to the ND group, which were downregulated by HRW administration (Figures 3A,C–E). Similarly, western blotting exhibited that increased protein expressions of α-SMA, FN and COL IV in kidneys caused by high oxalate diet were reduced with HRW administration (Figure 3F).

### RNA-seq Analysis and KEGG Pathway Analysis

**Figure 4:**
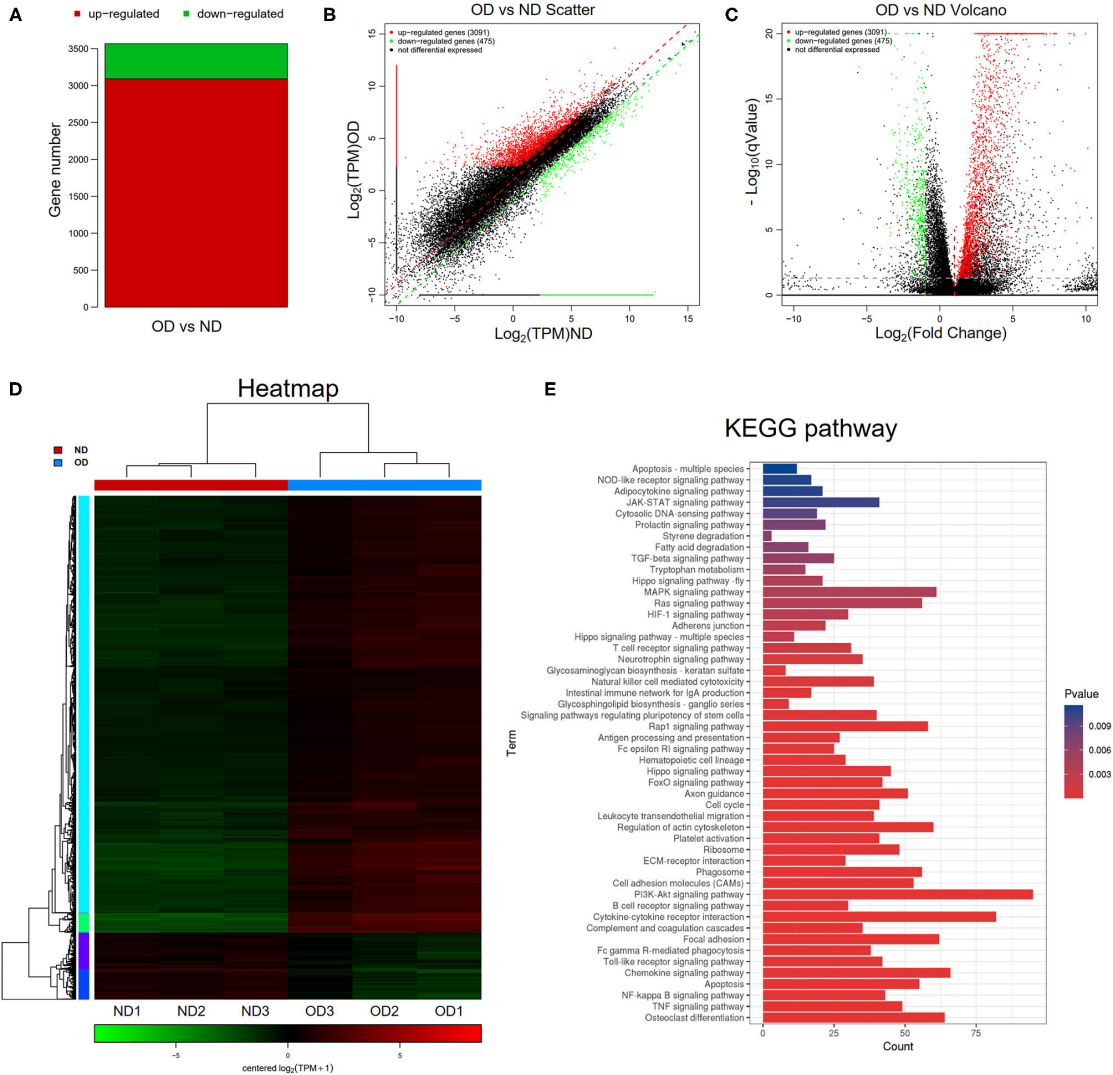
RNA-seq analysis and KEGG pathway analysis. **(A–C)** Histogram, scatter plot and volcano plot of DEGs between the OD and ND groups identified by differential expression analysis. **(D)** Heatmap and hierarchical clustering showing different gene expression patterns between the OD and ND groups. **(E)** The top 50 enriched KEGG pathways analysis of the identified DEGs.

RNA-seq analysis was performed to compare the gene expression profiles of renal tissues from the ND group and the OD group. DEGs were selected with the criteria of *q*-value <0.05, |log_2_FoldChange| > 1 and mean TPM of at least one group > 5. In total, 3,566 DEGs were selected and there were 3,091 upregulated and 475 downregulated DEGs between the OD and ND groups as shown in the histogram, scatter plot and volcano plot (Figures 4A–C). The different gene expression patterns between the OD and ND groups were indicted by the clustering heatmap (Figure 4D). All the identified DEGs were subjected to KEGG analysis by ClusterProfiler R package. KEGG analysis identified a variety of pathways in enrichment analysis, and the top 50 pathways were visualized (Figure 4E). PI3K/AKT signaling pathway, NF-κB signaling pathway and TGF-β signaling pathway were selected to further explore the effect of HRW administration on oxalate-induced renal injury.

### HRW Administration Suppresses Oxalate-Induced PI3K/AKT Signaling Pathway Activation in Mice

**Figure 5:**
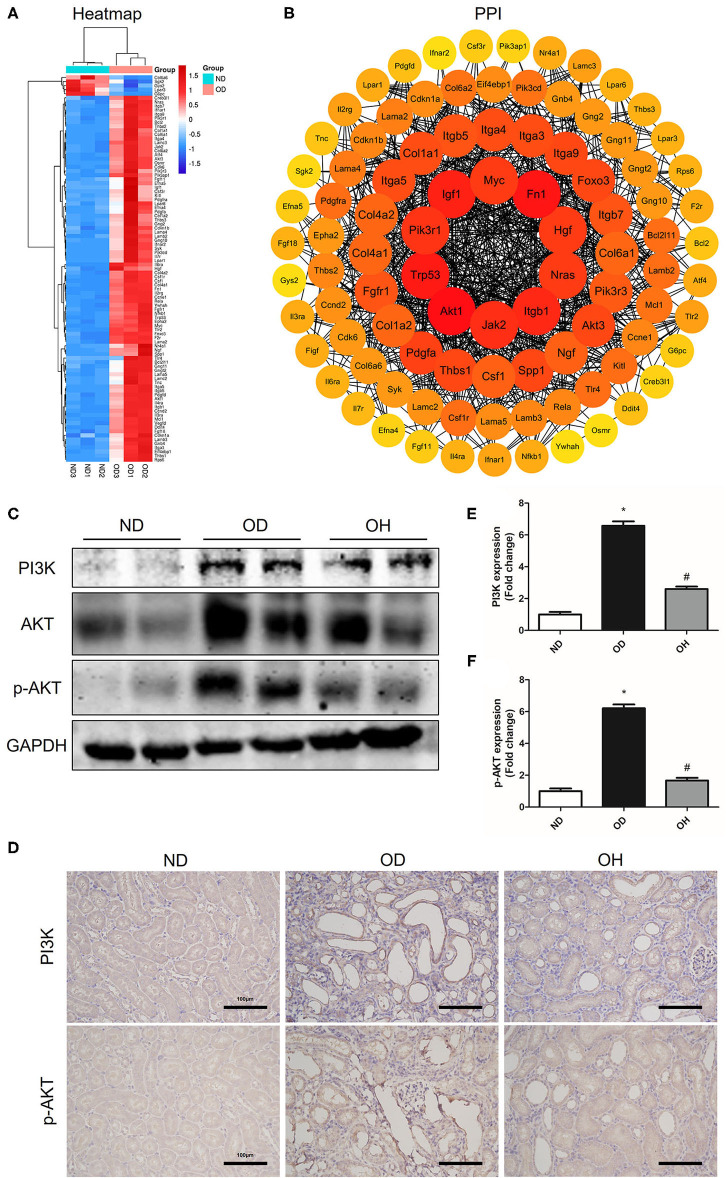
HRW administration suppresses oxalate-induced PI3K/AKT signaling pathway activation in mice. **(A)** Heatmap and hierarchical clustering showing DEGs enriched in the PI3K/AKT signaling pathway between the OD and ND groups. **(B)** PPI network constructed by DEGs enriched in the PI3K/AKT signaling pathway. **(C)** The protein expressions of PI3K, AKT, and p-AKT in kidneys of mice were detected by western blotting. **(D)** The protein expressions of PI3K and p-AKT in kidneys of mice were detected by immunohistochemistry (Scale bar = 100 μm). **(E,F)** Corresponding semiquantitative analysis of PI3K and p-AKT immunohistochemistry staining. The results were expressed as mean ± SEM. Statistical comparisons were performed using a NewmanKeuls test (*p < 0.05 vs. ND group, ^#^*p* < 0.05 vs. OD group).

In the PI3K/AKT signaling pathway, a total of 95 DEGs were enriched and there were 90 upregulated and 5 downregulated DEGs between the OD and ND groups as shown in the clustering heatmap (Figure 5A). Then, these 95 genes were entered into the STRING database to conduct a PPI network and 10 hub genes, including Akt1, Fn1, Trp53, Igf1, Itgb1, Jak2, Pik3r1, Nras, Hgf, and Myc, were found (Figure 5B). Afterward, western blotting was used to detect the expressions of key molecules in PI3K/AKT signaling pathway. As compared to the ND group, the protein expressions of PI3K, AKT, and p-AKT in kidneys of mice were enhanced in the OD group, which were downregulated by HRW administration (Figure 5C). Similarly, immunohistochemistry showed that increased expressions of PI3K and p-AKT in kidneys caused by high oxalate diet were reduced with HRW administration (Figures 5D–F).

### HRW Administration Suppresses Oxalate-Induced NF-κB Signaling Pathway Activation in Mice

**Figure 6:**
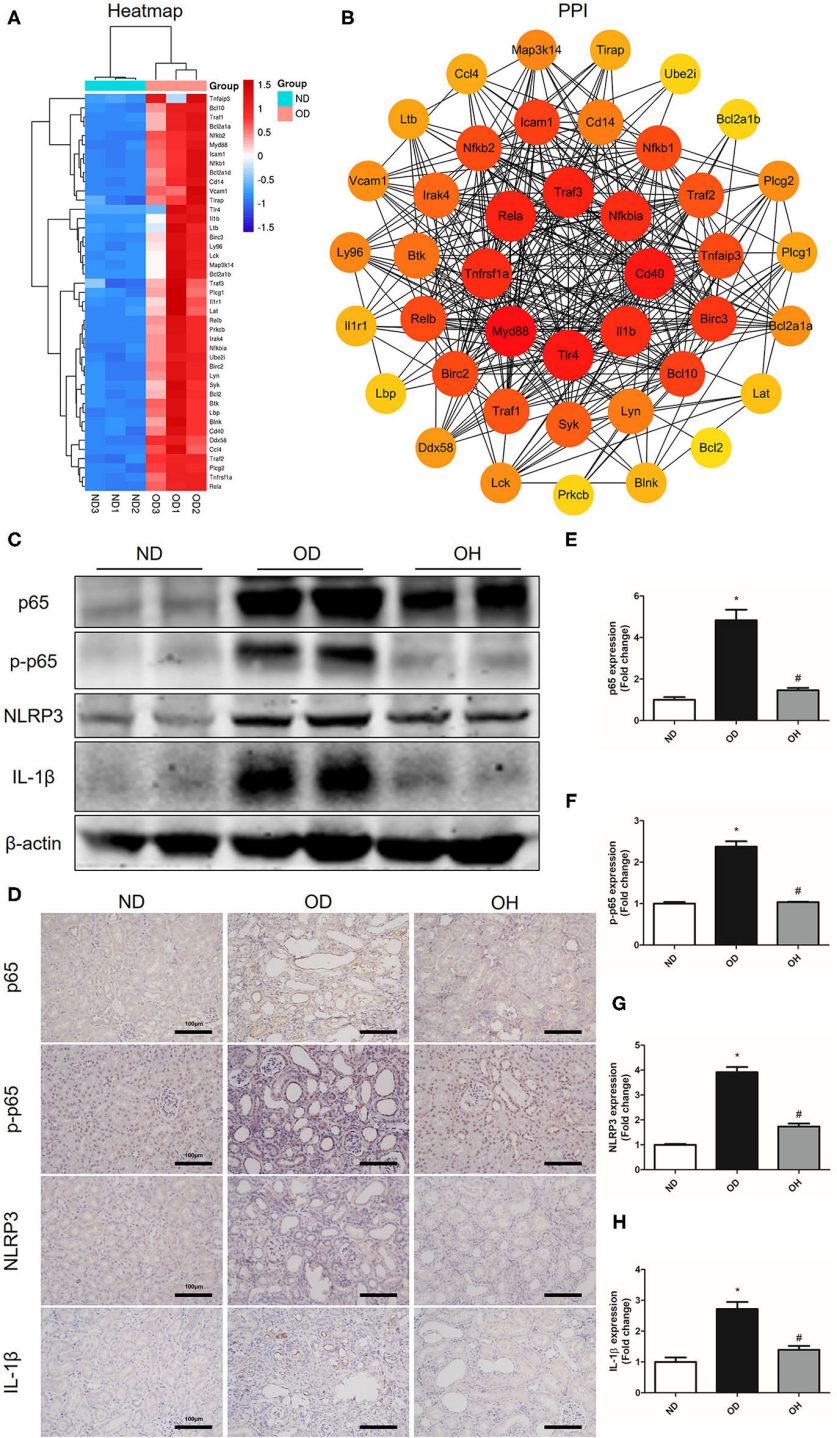
HRW administration suppresses oxalate-induced NF-κB signaling pathway activation in mice. **(A)** Heatmap and hierarchical clustering showing DEGs enriched in the NF-κB signaling pathway between the OD and ND groups. **(B)** PPI network constructed by DEGs enriched in the NF-κB signaling pathway. **(C)** The protein expressions of NF-κB p65, p-NF-κB p65, NLRP3 and IL-1β in kidneys of mice were detected by western blotting. **(D)** The protein expressions of NF-κB p65, p-NF-κB p65, NLRP3 and IL-1β in kidneys of mice were detected by immunohistochemistry (Scale bar = 100 μm). **(E–H)** Corresponding semiquantitative analysis of NF-κB p65, p-NF-κB p65, NLRP3, and IL-1β immunohistochemistry staining. The results were expressed as mean ± SEM. Statistical comparisons were performed using a NewmanKeuls test (^*^p < 0.05 vs. ND group, ^#^*p* < 0.05 vs. OD group).

As is shown in the clustering heatmap, 43 DEGs were enriched in NF-κB signaling pathway and these genes were all upregulated in the OD group compared to the ND group (Figure 6A). A PPI network was constructed with these 43 genes via the STRING database and 8 hub genes, including Myd88, Tlr4, Cd40, Traf3, Rela, Tnfrsf1a, Il1b, and Nfkbia, were found (Figure 6B). Then, the expressions of key molecules in NF-κB signaling pathway were examined by western blotting and immunohistochemistry. It was demonstrated that the expression levels of NF-κB p65, p-NF-κB p65, NLRP3, and IL-1β were higher in the OD group than the ND group. But the expression levels of these molecules were reversed in the OH group (Figure 6C). Immunohistochemistry also suggested that increased expressions of NF-κB p65, p-NF-κB p65, NLRP3, and IL-1β induced by high oxalate diet were suppressed with HRW administration (Figures 6D–H).

### HRW Administration Suppresses Oxalate-Induced TGF-β Signaling Pathway Activation in Mice

**Figure 7:**
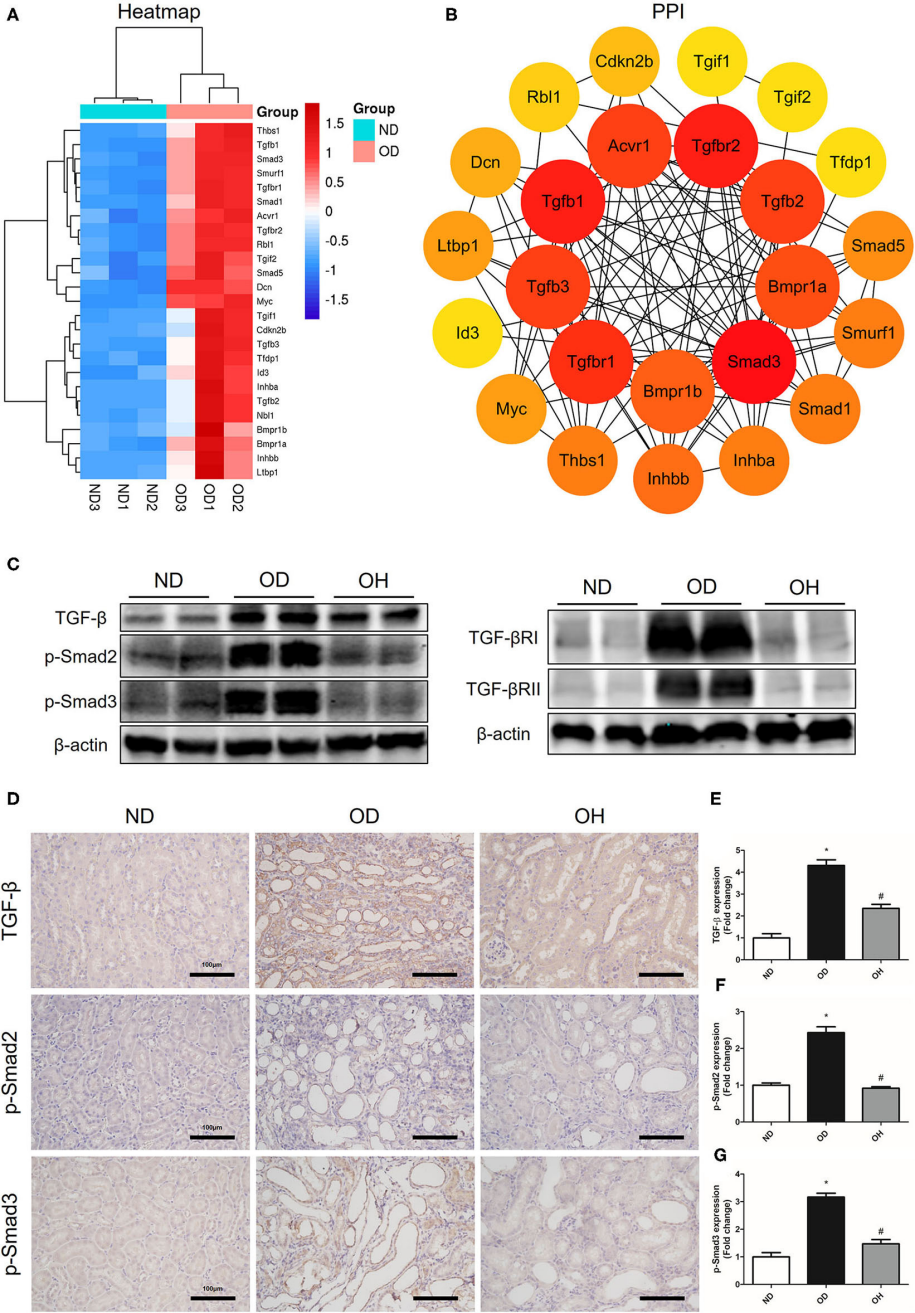
HRW administration suppresses oxalate-induced TGF-β signaling pathway activation in mice. **(A)** Heatmap and hierarchical clustering showing DEGs enriched in the TGF-β signaling pathway between the OD and ND groups. **(B)** PPI network constructed by DEGs enriched in the TGF-β signaling pathway. **(C)** The protein expressions of TGF-β, TGF-βRI, TGF-βRII, p-Smad2, and p-Smad3 in kidneys of mice were detected by western blotting. **(D)** The protein expressions of TGF-β, p-Smad2, and p-Smad3 in kidneys of mice were detected by immunohistochemistry (Scale bar = 100 μm). **(E–G)** Corresponding semiquantitative analysis of TGF-β, p-Smad2, and p-Smad3 immunohistochemistry staining. The results were expressed as mean ± SEM. Statistical comparisons were performed using a NewmanKeuls test (*p < 0.05 vs. ND group, ^#^*p* < 0.05 vs. OD group).

According to the clustering heatmap, a total of 25 DEGs were enriched in TGF-β signaling pathway and these genes were all upregulated in the OD group compared to the ND group (Figure 7A). Then, these 25 genes were entered into the STRING database to conduct a PPI network and 8 hub genes, including Smad3, Tgfbr2, Tgfb1, Tgfbr1, Tgfb3, Tgfb2, Acvr1, and Bmpr1a, were found (Figure 7B). After that, western blotting was used to detect the expressions of key molecules in TGF-β signaling pathway. The results showed that the expression levels of TGF-β, TGF-βRI, TGF-βRII, p-Smad2, and p-Smad3 were enhanced in the OD group compared to the ND group, which were downregulated with the administration of HRW (Figure 7C). Likewise, immunohistochemistry showed that increased expressions of TGF-β, p-Smad2, and p-Smad3 caused by high oxalate diet were reduced by HRW administration (Figures 7D–G).

## Discussion

Nephrolithiasis is a common disease associated with substantial prevalence and financial burden all over the world (1). Treatment options for patients with kidney stones include surgical stone removal and medication. Though instrument miniaturization has greatly improved the surgical treatment of kidney stones, the possible problems of surgical complications and high recurrence should not be ignored (14). Therefore, effective therapies for prevention and treatment of nephrolithiasis are strongly demanded. Previous studies showed that the pathophysiological mechanisms that lead to kidney stone formation involve oxidative stress, inflammation and fibrosis (PMID: 33514941). Molecular hydrogen can act as an important physiological regulatory factor to cells and organs on the anti-oxidative, anti-inflammatory, anti-fibrotic effects and other protective effects (8). In this study, we generated a mouse CaOx model with feeding a soluble oxalate diet and demonstrated that HRW administration attenuated CaOx crystal deposition and renal injury in mice.

It is established that oxidative stress resulted from ROS overproduction and decreased antioxidants is involved in CaOx crystal-induced renal tubular cell injury, which plays an important role in kidney stone formation (6, 7). In the present study, we found that HRW administration could alleviate oxidative stress induced by oxalate diet via decreasing the content of MDA and increasing the levels of SOD, GSH-Px and CAT. In addition, ROS production in renal tissues were also decreased with HRW administration. The overproduction of ROS can active the PI3K/AKT and NF-κB signaling pathways, which may further aggravate renal injury (15).

The PI3K/AKT pathway has been involved in many critical cellular functions, including protein synthesis, cell cycle progression, proliferation, apoptosis and autophagy (16). PI3K that activated by ROS and other conditions can catalyze the conversion of the membrane phospholipid phosphatidylinositol 4, 5-bisphosphate (PIP2) to phosphatidylinositol-3, 4, 5-triphosphate (PIP3). PIP3 then phosphorylates and activates AKT, which promotes the activation and transcription of downstream target genes, such as glycogen synthase kinase-3β (GSK-3β), mammalian target of rapamycin (mTOR), Snail and NF-κB (17–19). It has been suggested that the activation of PI3K/AKT could induce phosphorylation of GSK-3β and up-regulation of Snail, which could induce activation of fibroblast and accumulation of matrix production in the obstructed kidneys (20). A previous study found that the PI3K/AKT signaling pathway was activated in a CaOx mouse model with intragastric administration of glycol and inactivation of the PI3K/Akt pathway suppressed CaOx crystal deposition and the development of epithelial-mesenchymal transition (EMT) in kidneys (21). However, another study showed that miR-155 activated autophagy via repressing PI3K/Akt/mTOR signaling pathway, which facilitated CaOx crystal-induced renal tubular epithelial cell injury (22). In our study, we found that PI3K/AKT pathway was activated by oxalate diet, but suppressed by HRW. Therefore, we speculate that HRW may ameliorate oxalate-induced kidney injury via inhibiting oxidative stress-mediated PI3K/AKT signaling.

The transcription factor NF-κB is crucial in a series of cellular processes, including inflammation, cellular adhesion, immunity, differentiation, proliferation and apoptosis (23). NF-κB pathway is triggered in response to oxidative stress and other conditions, then NF-κB translocates into the nucleus and promotes the transcription of TNF-α, IL-1β and Monocyte chemotactic protein 1 (MCP-1) (24). NLRP3 inflammasome plays a critical role in the maturation of IL-1β and inflammation. On one hand, NLRP3 inflammasome can accelerate NF-κB activation and promote NF-κB pathway-mediated inflammatory response (25). On the other hand, NF-κB can in turn promote the transcription of NLRP3 via binding to the NLRP3 promoter (26). Previous studies suggested that NF-κB and NLRP3 inflammasome pathways were activated and inflammatory cytokine production was increased in CaOx crystal-induced kidney injury. Besides, suppression of NF-κB and NLRP3 inflammasome could decrease the release of inflammatory mediators and ameliorate CaOx crystal-induced renal inflammation (27, 28). Furthermore, it has been demonstrated that oxalate induced OPN expression by activating NF-κB in renal tubular cells (29). OPN, as the main organic component of the stone matrix, promotes the adhesion of CaOx crystals to surface of tubular cells via binding to its receptor CD44 (30). In the present study, we found that the levels of TNF-α and IL-1β in serum were up-regulated, the expressions of OPN and CD44 in renal tissues were increased, and the NF-κB pathway was activated in the mice fed with high oxalate diet. These situations were improved by HRW administration. Thus, we assume that HRW administration can alleviate oxalate-induced renal inflammation by suppressing the NF-κB signaling pathway.

Excessive oxidative stress and inflammation in the progress of stone formation inevitably promote EMT of tubular cells and renal fibrosis (31). TGF-β is a profibrotic cytokine found in renal diseases, which induces renal cells to produce extracellular matrix proteins leading to tubulointerstitial fibrosis (32). TGF-β/Smad signaling is the main pathway in progressive renal fibrosis. It has been suggested that Smad2 and Smad3 were activated in the fibrotic kidneys of CKD patients and animal models (33). Similarly, our previous study reported that TGF-β/Smad pathway was activated in a CaOx mouse model with intraperitoneal injection of glyoxylate, which induced EMT and renal fibrosis (34). In this study, we found that the content of collagen and expressions of fibrogenic cytokines were increased, and TGF-β/Smad pathway was activated by oxalate diet. HRW administration could improve these conditions. Hence we guess that HRW can ameliorate oxalate-induce renal fibrosis via inhibiting the TGF-β/Smad signaling pathway.

**Figure 8:**
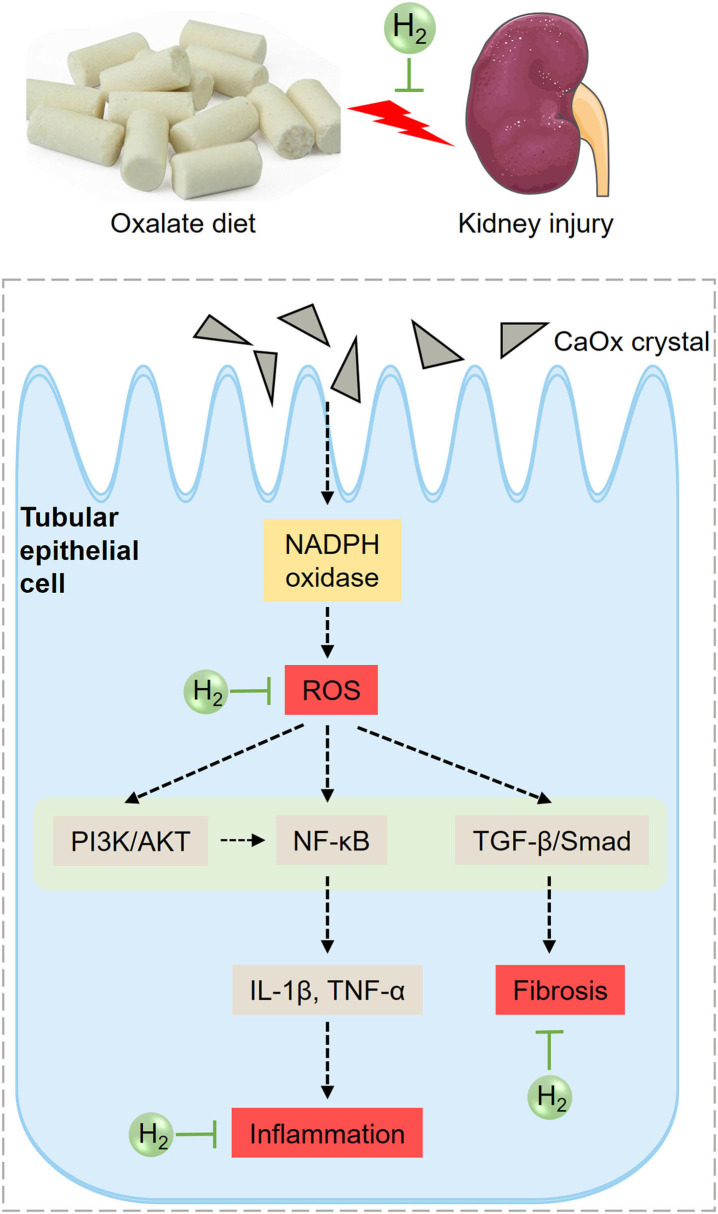
Diagram of the effects of HRW on oxalate-induced kidney injury. The increased CaOx crystals induced by high oxalate diet activate NADPH oxidase and enhance renal ROS production. ROS can further activate PI3K/AKT, NF-κB, and TGF-β pathways, increasing the production of inflammatory factors and collagen deposition, promoting inflammation and fibrosis. HRW may alleviate oxalate-induced kidney injury through improving oxidative stress, inflammation and fibrosis via suppressing PI3K/AKT, NF-κB, and TGF-β signaling pathways.

Based on the above analysis, the potential mechanism of HRW on oxalate-induced kidney injury was summarized (Figure 8). The increased CaOx crystals induced by high oxalate diet activate NADPH oxidase and enhance renal ROS production. ROS can further activate PI3K/AKT, NF-κB and TGF-β pathways, increasing the production of inflammatory factors and collagen deposition, promoting inflammation and fibrosis. HRW may alleviate oxalate-induced kidney injury through improving oxidative stress, inflammation and fibrosis via suppressing PI3K/AKT, NF-κB, and TGF-β signaling pathways.

## Data Availability Statement

The datasets presented in this study can be found in online repositories. The names of the repository/repositories and accession number(s) can be found below: https://www.ncbi.nlm.nih.gov/, PRJNA743727.

## Ethics Statement

The animal study was reviewed and approved by Committee on Ethic of Medical Research, Naval Medical University (registration number: NMUMREC-2021-006).

## Author Contributions

ZG, HL, and XS conceived and designed the experiments. YS, LL, JC, TZ, QZ, and JY performed the experiments. YS and LL analyzed the data and drafted the manuscript. WC, JD, XS, HL, and ZG revised the manuscript. All authors contributed to the article and approved the submitted version.

## Conflict of Interest

The authors declare that the research was conducted in the absence of any commercial or financial relationships that could be construed as a potential conflict of interest.

## Publisher's Note

All claims expressed in this article are solely those of the authors and do not necessarily represent those of their affiliated organizations, or those of the publisher, the editors and the reviewers. Any product that may be evaluated in this article, or claim that may be made by its manufacturer, is not guaranteed or endorsed by the publisher.
